# Effects of sex and age on the association of relational aggression with mental health problems and functional impairment in adolescents

**DOI:** 10.3389/fpsyg.2026.1800051

**Published:** 2026-06-09

**Authors:** Roman Koposov, Johan Isaksson, Andrew Stickley, Denis G. Sukhodolsky, Vladislav Ruchkin

**Affiliations:** 1 Regional Centre for Child and Youth Mental Health and Child Welfare, Northern Norway, UiT The Arctic University of Norway, Tromsø, Norway; 2 Sechenov First Moscow State Medical University, Moscow, Russia; 3 Child and Adolescent Psychiatry Unit, Department of Medical Sciences, Uppsala University, Uppsala, Sweden; 4 Center of Neurodevelopmental Disorders, Centre for Psychiatry Research, Department of Women’s and Children’s Health, Karolinska Institutet and Stockholm Health Care Services, Region Stockholm, Stockholm, Sweden; 5 Department of Preventive Intervention for Psychiatric Disorders, National Institute of Mental Health, National Center of Neurology and Psychiatry, Kodaira, Tokyo, Japan; 6 Child Study Center, Yale School of Medicine, New Haven, CT, United States

**Keywords:** adolescents, functional impairment, mental health problems, posttraumatic stress disorder, relational aggression, sex

## Abstract

**Introduction:**

Relational aggression (RA), characterized by behaviors aimed at inflicting social harm, is prevalent during adolescence and linked to adverse mental health outcomes. Although RA has been associated with internalizing and externalizing problems, less is known about its relationship with functional impairments (i.e., a reduced ability to perform daily roles) and sex and age differences. This study aimed to examine the associations between RA and mental health outcomes and functional impairment, and to test the effects of sex and age on these associations.

**Methods:**

Data were used from a representative sample of 2,838 adolescents aged 13–17 years from Arkhangelsk, Russia. Participants completed validated self-report measures assessing RA, depressive symptoms, anxiety, somatic complaints, posttraumatic stress, conduct problems, alcohol use, and functional impairment. Multivariate path analyses were used to estimate associations between RA and all outcomes simultaneously, adjusting for sex, age, and a proxy measure of socioeconomic status (SES).

**Results:**

RA was significantly associated with depressive symptoms, somatic complaints, posttraumatic stress, conduct problems, alcohol use, and functional impairment, but not anxiety after accounting for the substantial intercorrelations among symptom domains. Sex moderated these associations, with boys showing greater increases in internalizing symptoms and girls exhibiting higher conduct problems and alcohol use at higher levels of RA. Age and the SES proxy measure were also significant predictors of mental health outcomes. No RA-by-age interactions were observed.

**Discussion:**

By modeling multiple outcomes simultaneously, this study provides novel evidence that RA is associated with a broad and distinctive pattern of psychosocial difficulties beyond general internalizing or externalizing distress. Findings suggest that RA is a clinically relevant marker of adolescent vulnerability with sex-specific correlates that may inform targeted prevention efforts.

## 1 Introduction

Relational aggression (RA) refers to behaviors intended to inflict social harm through the manipulation of peer relationships, such as excluding others, withdrawing friendship or attention, spreading rumors or gossip, or publicly humiliating peers (Crick and Grotpeter, 1995; Steinberg, 2008; Archer, 2009). These behaviors may be enacted overtly or covertly, occur in face-to-face or online contexts, and serve either reactive functions (e.g., retaliation to perceived slights) or proactive, instrumental goals (e.g., gaining social leverage, dominance, or status within the peer group) (Fite et al., 2009). Although engagement in RA can confer short-term interpersonal advantages, such as increased perceived popularity or influence, it is also associated with substantial longer-term psychosocial costs for both perpetrators and targets (Rose et al., 2004; Card et al., 2008).

From a social information-processing perspective, RA can be understood as an interpersonal strategy that emerges from how adolescents interpret social cues, evaluate peer-group goals, and select behavioral responses in socially ambiguous or threatening situations. Social information-processing models propose that aggressive behavior is shaped by sequential processes, including the encoding and interpretation of social cues, clarification of goals, generation and evaluation of possible responses, and enactment of selected behaviors (Crick and Dodge, 1994; Crick and Dodge, 1996). Applied to RA, adolescents who perceive relational threat, exclusion, or status competition may select indirect forms of aggression as a means of restoring control, protecting social position, or retaliating against perceived social harm. Later refinements of this model emphasize that affective arousal and emotion regulation are integral to social-cognitive processing, suggesting that RA may reflect both instrumental social goals and difficulties in managing interpersonal stress (Lemerise and Arsenio, 2000).

RA is particularly prevalent during adolescence, a developmental period marked by heightened sensitivity to peer evaluation, increasing social complexity, and the growing importance of peer group status (Crick and Grotpeter, 1995; Underwood, 2003). In an earlier study 16.8 and 18.4% of students were classified as perpetrators and victims of RA, respectively (Williams et al., 2009). A large body of research has consistently linked higher levels of RA to a range of adverse mental health outcomes. Adolescents who engage in RA report elevated internalizing difficulties, including depressive symptoms, anxiety, loneliness, and social withdrawal, as well as increased externalizing problems such as conduct difficulties, delinquency, and attentional problems (Zimmer-Gembeck et al., 2005; Card et al., 2008). However, despite substantial evidence linking RA to mental health problems, less is known about the association between RA and functional impairment, commonly defined as a measurable reduction in the ability to perform age-appropriate daily activities due to physical, mental, or emotional conditions (Merrell et al., 2006; Üstün and Kennedy, 2009). Moreover, existing research has largely examined these outcomes in isolation, using bivariate or single-outcome models, limiting understanding of whether RA exerts unique associations with specific symptom domains once co-occurring difficulties are taken into account.

Stress and coping theory provide a complementary framework for understanding why RA may be associated with broad mental health difficulties and functional impairment. According to transactional models of stress, psychological outcomes depend not only on exposure to stressful events but also on how individuals appraise, respond to, and cope with those events (Lazarus and Folkman, 1984). RA is likely to occur within peer contexts characterized by social uncertainty, conflict, reputational threat, retaliation, and exclusion. These experiences may function as chronic interpersonal stressors that increase emotional arousal, undermine perceived social safety, and contribute to internalizing symptoms, somatic complaints, posttraumatic stress symptoms, and impaired functioning. At the same time, RA itself may represent a maladaptive coping or regulation strategy, particularly when adolescents use relational manipulation to manage anger, jealousy, insecurity, or perceived loss of social control. Thus, RA may be both a behavioral expression of interpersonal stress and a contributor to further stress exposure.

Importantly, associations between RA and mental health vary by sex, developmental stage, and cultural context. Although early work suggested sex-specific patterns of aggression, with boys exhibiting more physical aggression and girls more RA (Crick and Grotpeter, 1995), later research indicates that gender differences in RA are often small or inconsistent, challenging the view of RA as a uniquely female form of aggression (Archer, 2004; Voulgaridou and Kokkinos, 2015). Developmentally, RA in early adolescence may appear socially strategic and be linked to higher perceived popularity despite lower likeability, masking distress, whereas by mid-to-late adolescence it more consistently predicts depression, anxiety, loneliness, friendship instability, and, particularly among boys, rule-breaking behavior (Zimmer-Gembeck et al., 2005; Card et al., 2008). Cross-cultural findings further demonstrate variability in the sex patterns of RA, emphasizing the influence of social norms rather than true mean-level differences (Murray-Close et al., 2007; Voulgaridou and Kokkinos, 2023). Studying RA in Russian adolescents is therefore especially relevant given reports of different cultural norms from those observed in the West, high social stress exposure, and elevated adolescent alcohol use (Hofstede, 2001; Inchley et al., 2018; World Health Organization, 2020; Volkova, 2025).

A developmental psychopathology perspective further suggests that RA should be examined not as an isolated behavior but as part of a broader pattern of adaptation and maladaptation across interconnected developmental systems. Developmental psychopathology emphasizes that peer relationships, emotional regulation, behavioral control, school functioning, and mental health are mutually influential over time, and that difficulties in one domain may cascade into other areas of functioning (Masten and Cicchetti, 2010; Murray-Close et al., 2016). Within this framework, RA may contribute to psychopathology through multiple, overlapping pathways: by disrupting peer relationships, increasing exposure to interpersonal stress, reinforcing maladaptive social goals, and weakening adolescents’ capacity to function effectively in school, family, leisure, and peer contexts. This perspective supports the need to examine multiple mental health outcomes and functional impairment simultaneously rather than treating each outcome as independent.

Guided by these complementary theoretical perspectives, the present study conceptualized RA as a socially embedded behavior that may reflect maladaptive social-cognitive processing, chronic interpersonal stress, and developmental risk for broader psychosocial impairment. Specifically, social information-processing theory suggests that RA may be linked to hostile interpretations of peer behavior, status-oriented goals, and aggressive response selection; stress and coping theory suggests that RA may both arise from and intensify interpersonal stress; and developmental psychopathology highlights the possibility that RA is associated with difficulties across multiple domains of adjustment. Accordingly, we hypothesized that higher RA would be associated with greater internalizing symptoms, externalizing problems, alcohol use, posttraumatic stress symptoms, somatic complaints, and functional impairment, even after accounting for sex, age, and socioeconomic status (SES) (Yang et al., 2026), and intercorrelations among outcomes. Given prior evidence that the expression and correlates of RA may differ by sex and developmental stage, we further examined whether these associations were moderated by sex and age.

## 2 Materials and methods

### 2.1 Participants and procedure

The study was carried out in the largest city in Northwestern Russia, Arkhangelsk. According to official statistics (Russian Federal State Statistics Service, 2011), the city has a population of just over 346,000, including roughly 30,000 adolescents aged 13–17. A randomized sampling procedure was employed to obtain a representative school-based sample, with schools and classes serving as the units of randomization. In the first stage, 14 schools were randomly selected from a total of 71 eligible schools, encompassing 210 classes across grades 6–11. In the second stage, data were collected from students in 70 randomly chosen classes, resulting in an initial sample of 2,892 students, corresponding to a 96.4% participation rate.

Participants outside the targeted age range of 13–17 years (*n* = 45) and those lacking data on sex (*n* = 9) were excluded, resulting in a final analytic sample of 2,838 adolescents. For participants missing data on continuous variables (*n* = 300), multiple imputation using the expectation–maximization (EM) algorithm was conducted in SPSS (Version 30), with a maximum of 25 iterations. Across study variables, missing data ranged from 0 to 5.1% (Little’s MCAR test: *χ*^2^ = 525.43, *p* < 0.001). Adolescents with incomplete data were more likely to be male (54.0% vs. 40.7%; *χ*^2^(1) = 19.58, *p* < 0.001) and reported slightly lower levels of anxiety [M (SD) = 13.37 (5.68) vs. 12.18 (5.50); *t* = 3.02, *p* < 0.01] and posttraumatic stress [M (SD) = 20.01 (11.44) vs. 18.03 (12.43); *t* = 2.77, *p* < 0.01]. The excluded youth did not differ from included participants on any other study variable. Although Little’s MCAR test was significant, missingness was low across variables, and EM imputation was selected to preserve statistical power and reduce bias under conditions consistent with data missing at random.

The final sample consisted of 2,838 participants [M (SD) age = 14.89 (1.12)], of whom 57.9% were female (*n* = 1,644). The sample was predominantly of Russian origin (95.8%), with small proportions of Ukrainian, Belarusian, and other nationalities, closely reflecting the demographic composition of the local public school population. Most participants (75.8%) lived in two-parent households, while 24.2% had divorced, separated, or widowed parents. According to student reports, 93.1% of fathers and 94.4% of mothers had completed at least a high school education.

Parents were initially notified about the survey and given the option to decline their child’s participation. Informed consent was obtained from parents and/or legal guardians. Before the survey was administered, students were presented with a detailed assent form explaining their participation, assured of confidentiality, and asked to sign the form to indicate their assent. Students were also given the option to decline participation at the time of the survey (less than 1% of parents and students opted out). The survey was completed by students during a single class period (45 min) on a regular school day and was conducted in Russian. Ethical approval for the study was granted by the institutional review committee and the scientific council of the Institute of Psychiatry at the Northern State Medical University (Arkhangelsk, Russia). Additionally, permission to conduct the study was obtained from the Arkhangelsk city administration.

### 2.2 Measures

For each measure, we report the assessment name or source, number of items, response format, possible score range, and interpretation of higher scores.

*Relational aggression* (Ruchkin et al., 2026) was assessed with 10 items describing the respondent’s RA toward a peer they did not like. The students were provided with the introductory statement: “There are people with whom we really enjoy spending time and want to communicate, and there are those whom we do not like at all. Everyone reacts differently to people they don’t like. Please indicate how you acted toward a peer you didn’t like.” Items were rated using a 4-point scale ranging from “Almost never” (1) to “Almost always” (4). The possible total score ranged from 10 to 40, with higher scores indicating more RA. This instrument has been used with Russian adolescents previously, where, similar to earlier studies (Card et al., 2008), RA was found to represent a distinct factor, different from physical and verbal aggression (Isaksson et al., 2020). Cronbach’s *α* for the scale was 0.83.

*Depressive symptoms* were assessed using an adapted version of the Center for Epidemiologic Studies Depression Scale (CES-D) (Radloff, 1977), which has demonstrated strong psychometric properties in adolescent samples (Carpenter et al., 1998) and has been used previously with Russian adolescents (Ruchkin et al., 2023). Participants reported on the presence of 10 depressive symptoms during the past 30 days on a 3-point scale (“Not true” = 0, “Somewhat true” = 1, “Certainly true” = 2). Scores ranged from 0 to 20, with higher scores indicating greater levels of depressive symptoms. The internal consistency of this measure was good (Cronbach’s *α* = 0.84).

*Somatic complaints* were measured using a nine-item scale assessing physical symptoms commonly reported by children and adolescents (Taylor et al., 1996). Items assess symptoms experienced during the past month and included headaches, health-related worries, stomach aches, general aches or pains, nausea, rashes or skin problems, not feeling well, and vomiting. Responses were rated on a 3-point scale (“Not true” = 0, “Somewhat true” = 1, “Certainly true” = 2). Total scores ranged from 0 to 18, with higher scores indicating greater levels of somatic problems. This measure has been used previously with Russian adolescents (Tingstedt et al., 2018) and had good internal consistency in the current study (Cronbach’s *α* = 0.83).

*Anxiety symptoms* were assessed with a 12-item scale measuring worrisome thoughts and avoidance behaviors (Ruchkin et al., 2004). Items (e.g., “I feel nervous when I get called on in class,” “I stay away from things that make me nervous”) were rated on a 3-point scale ranging from “Not true” (0) to “Certainly true” (2). This scale has been widely used with Russian adolescents (e.g., Schwab-Stone et al., 2013). Total scores ranged from 0 to 24, with higher scores indicating greater anxiety symptoms (Cronbach’s *α* = 0.83).

*Posttraumatic stress* symptoms were assessed using the Child Post-Traumatic Stress Reaction Index (CPTS-RI) (Steinberg et al., 2004). The scale includes 20 items assessing the frequency of trauma-related symptoms over the past month on a 5-point scale (“Never” = 0 to “Most of the time” = 4). Total scores range from 0 to 80 and correspond closely with clinical posttraumatic stress disorder (PTSD) severity categories (Pynoos et al., 1993). The CPTS-RI has demonstrated validity in Russian adolescent samples (Ruchkin et al., 2002; Saunderson et al., 2022). Internal consistency in the present sample was high (Cronbach’s α = 0.87).

*Conduct problems* were measured using a six-item scale assessing relatively mild problem behaviors (Schwab-Stone et al., 1999). Participants reported how often they engaged in behaviors such as lying to adults, staying out overnight without permission, shoplifting, damaging property, and skipping school during the past year, using a 5-point frequency scale (“0 times” to “5 or more times”). Total scores ranged from 0 to 24, with higher scores indicating increased conduct problems (Cronbach’s *α* = 0.79).

*Alcohol use* was assessed using four items adapted from the Monitoring the Future survey (Johnston et al., 2016). Participants reported their frequency of beer, wine, and strong alcohol consumption during the past 30 days on a 4-point scale (“Never” = 0 to “More than a few times” = 3), as well as if they drank five or more drinks in a row in the same time period on a 5-point scale. Total scores ranged from 0 to 13, with higher scores indicating greater alcohol use.

*Functional impairment* was assessed using the Impact Supplement of the Strengths and Difficulties Questionnaire (SDQ) (Goodman, 1997), which has been validated in Russian samples (Ruchkin et al., 2007). The supplement assesses perceived subjective distress related to difficulties in emotional, behavioral, and social areas and interference in home life, friendships, classroom learning, and leisure activities, as well as the perceived burden on others. Responses range from “Not at all” (0) to “A great deal” (2). If participants indicated no difficulties on the initial screening item, the impact score was set to zero. Total scores were calculated by summing up the five items. The internal consistency of this measure was acceptable (Cronbach’s α = 0.78).

A *proxy measure of socioeconomic status* (SES) was constructed using adolescents’ reports of parental education and employment status. For each parent, completion of a college degree or higher was scored as 1, and current employment was scored as 1, yielding a total household score ranging from 0 to 4. For single-parent households, the score was divided by two to adjust for lower potential maximum values. This SES index proxy measure has been used in prior research (Löfving-Gupta et al., 2015).

### 2.3 Statistical analyses

SPSS version 30.0 and RStudio version 2025.09.0 + 387 were used for all analyses. Descriptive statistics were computed for all study variables, including means, standard deviations, skewness, and kurtosis. Spearman’s rho correlations were computed to examine bivariate associations among the study variables. Spearman’s rho was selected because several variables were non-normally distributed and positively skewed, particularly RA and functional impairment, and because several measures were bounded or ordinal in nature. To account for multiple testing in the correlation matrix, *p*-values were adjusted using the Benjamini–Hochberg false discovery rate procedure across the 55 unique correlations.

Primary analyses were conducted using full path analysis in R using the lavaan package with robust maximum likelihood estimation. These models assessed associations between RA and the psychopathology outcomes, including depression, anxiety, somatic complaints, posttraumatic stress, conduct problems, alcohol use, and functional impairment, while adjusting for sex, age, the SES proxy variable, and the intercorrelations among the predictors and outcomes. Since all continuous variables were positively skewed, we also applied a square root transformation to decrease the skewness in the path analytic model. These results are presented in Supplementary Table S1.

Given the observed sex differences in several of the study variables, sex was included as a covariate in the path model. In addition, RA-by-sex interaction terms were tested to examine whether associations between RA and the outcomes differed between boys and girls. This full-sample moderation approach was selected rather than estimating separate models by sex because it allowed direct statistical tests of sex differences in the associations to be undertaken while retaining statistical power. RA-by-age interaction terms were also tested to examine whether associations differed by age. Nonsignificant interactions were removed from the final model.

Multiple-comparison correction was not applied to the path model, as parameters were estimated simultaneously within a theory-driven structural equation modeling framework and interpreted in the context of the overall model. Results are reported as means and standard deviations, and standardized regression coefficients were used to assess the strength of associations. Two-tailed tests with *p* < 0.05 were considered statistically significant.

## 3 Results

Descriptive statistics of all the study variables are presented in Table 1. Several variables had a positive skew, including RA, posttraumatic stress symptoms, conduct problems, alcohol use, and functional impairment. Functional impairment showed the strongest skewness and kurtosis, consistent with the expectation that many adolescents report low levels of impairment in population-based samples.

**Table 1:** Descriptive statistics of the study variables.

Variable	*n*	Mean	SD	Skewness	Kurtosis
Depression	2,838	5.97	4.18	0.64	−0.04
Anxiety	2,838	13.28	5.61	−0.02	−0.42
Somatic symptoms	2,838	5.14	3.63	0.65	0.02
Posttraumatic stress	2,838	19.81	11.53	1.10	1.42
Conduct problems	2,838	4.69	4.72	0.97	0.19
Alcohol use	2,838	3.23	3.47	1.07	0.23
Functional impairment	2,838	0.85	1.58	2.48	6.90
Relational aggression	2,838	15.10	4.82	1.41	3.72
SES proxy measure	2,838	2.10	1.10	0.21	−0.81
Age	2,838	14.89	1.12	0.19	−0.73

SD, standard deviation; SES, socioeconomic status. Skewness and kurtosis values are reported to describe the distributional characteristics of the study variables. Standard errors were 0.046 for skewness and 0.092 for kurtosis for all variables.

### 3.1 Associations between the study variables

Bivariate correlations among the study variables are presented in Table 2. RA was moderately correlated with conduct problems and posttraumatic stress symptoms and showed weak to moderate correlations with depressive symptoms, anxiety, somatic complaints, alcohol use, functional impairment, and age. The strongest correlations overall were observed between depressive symptoms and posttraumatic stress, and between depressive symptoms and somatic complaints. Girls reported higher levels of depressive symptoms, anxiety, somatic complaints, posttraumatic stress, and functional impairment, while boys reported higher levels of conduct problems. Sex was not correlated with RA. Multiple-comparison correction was applied across the 55 unique bivariate correlations reported in the correlation matrix using the Benjamini–Hochberg false discovery rate procedure. All associations remained significant.

**Table 2:** Spearman rank-order correlations among RA, mental health outcomes, substance use, functional impairment, and covariates.

Variable	Depression	Anxiety	Somatic symptoms	Posttraumatic stress	Conduct problems	Alcohol use	Functional impairment	Gender	Age	SES proxy measure
Relational aggression	0.21***	0.11***	0.15***	0.28***	0.37***	0.23***	0.23***	−0.02	0.06**	−0.00
Depression	–	0.36***	0.47***	0.56***	0.21***	0.10***	0.44***	−0.19***	0.12***	−0.03
Anxiety		–	0.27***	0.37***	−0.05*	−0.13***	0.24***	−0.15***	−0.03	−0.03
Somatic symptoms			–	0.37***	0.16***	0.06**	0.28***	−0.14***	0.06**	−0.02
Posttraumatic stress				–	0.17***	0.09***	0.43***	−0.19***	0.06**	−0.04*
Conduct problems					–	0.45***	0.20***	0.07***	0.20***-	0.00
Alcohol use						–	0.08***	0.02	0.25***	0.03
Functional impairment							–	−0.09***	0.08***	−0.02
Gender								–	−0.08***	−0.03
Age									–	0.05**

RA, Relational aggression; SES, socioeconomic status, ***p < 0.001; **p < 0.01; *p < 0.05. Names of specific assessments are provided in the Measures section. Spearman’s rho correlations are reported. *p*-values were adjusted for multiple comparisons using the Benjamini–Hochberg false discovery rate (FDR) procedure across the 55 unique correlations in the matrix. All statistically significant associations remained significant after FDR correction.

### 3.2 Full path analytic model

Results of the full path model are presented in Table 3. The model explained between 4 and 15% of the variance in the outcome variables. Multiple-comparison correction was not applied to the path model, as parameters were estimated simultaneously and interpreted in the context of the overall model. RA was associated with significantly higher levels of depression, *β* = 0.15, somatic symptoms, *β* = 0.10, posttraumatic stress, *β* = 0.21, conduct problems, *β* = 0.35, alcohol use, *β* = 0.25, and functional impairment, *β* = 0.21. RA was not significantly associated with anxiety, *β* = 0.05, after adjustment for the other variables in the model. The strongest association was observed for conduct problems, followed by alcohol use, posttraumatic stress, and functional impairment.

**Table 3:** Path analytic model of associations between psychopathology and RA, age, and sex, with standardized regression coefficients (*β*).

Parameter estimated	Depression	Anxiety	Somatic symptoms	Posttraumatic stress	Conduct problems	Alcohol use	Functional impairment
	*β* (95% CI)	*β* (95% CI)	*β* (95% CI)	*β* (95% CI)	*β* (95% CI)	*β* (95% CI)	*β* (95% CI)
RA	0.15 (0.10, 0.21)***	0.05 (−0.00, 0.10)	0.10 (0.05, 0.16)***	0.21 (0.15, 0.27)***	0.35 (0.30, 0.40)***	0.25 (0.20, 0.31)***	0.21 (0.15, 0.26)***
Sex (male)	−0.38 (−0.50, −0.26)***	−0.33 (−0.44, 0.21)***	−0.30 (−0.43, −0.17)***	−0.43 (−0.55, −0.30)***	0.13 (0.01, 0.25)*	0.21 (0.09, 0.33)**	−0.14 (−0.26, −0.02)*
Age	0.10 (0.06, 0.13)***	−0.04 (−0.08, −0.00)*	0.04 (0.01, 0.08)*	0.02 (−0.01, 0.06)	0.18 (0.15, 0.22)***	0.21 (0.17, 0.24)***	0.06 (0.03, 0.10)**
SES proxy measure	−0.05 (−0.08, −0.01)**	−0.03 (−0.07, 0.00)	−0.02 (−0.06, 0.01)	−0.06 (−0.09, −0.03)**	−0.01 (−0.04. 0.03)	0.02 (−0.01, 0.06)	−0.05 (−0.08, −0.01)**
RA × sex	0.23 (0.09, 0.36)**	0.18 (0.06, 0.30)**	0.20 (0.06, 0.34)**	0.29 (0.15, 0.44)***	−0.05 (−0.18, 0.08)	−0.17 (−0.30, −0.04)*	0.09 (−0.06, 0.23)
*R* ^2^	0.10	0.04	0.05	0.12	0.15	0.09	0.07

RA, Relational aggression; SES, socioeconomic status; CI, confidence interval; *R*^2^, coefficient of determination. ***p < 0.001; **p < 0.01; *p < 0.05. Names of specific assessments are provided in the measures section.

Sex was independently associated with all outcomes. Compared with girls, boys reported lower levels of depression, anxiety, somatic symptoms, posttraumatic stress, and functional impairment, but higher levels of conduct problems and alcohol use. Age was positively associated with depression, somatic symptoms, conduct problems, alcohol use, and functional impairment, and negatively associated with anxiety. The proxy measure of SES was negatively associated with depression, posttraumatic stress, and functional impairment.

No significant interaction effects were observed between RA and age and these results were therefore removed from the final model. Significant interaction effects between RA and sex were observed for depression, *β* = 0.23, anxiety, *β* = 0.18, somatic symptoms, *β* = 0.20, posttraumatic stress, *β* = 0.29, and alcohol use, *β* = −0.17. These interactions indicated that the associations between RA and depression, anxiety, somatic symptoms, and posttraumatic stress were stronger among boys, whereas the association between RA and alcohol use was stronger among girls. No significant RA-by-sex interactions were observed for conduct problems or functional impairment.

In addition, after applying the square root transformation to the skewed variables, the same pattern of associations in the path analytic model emerged, with RA being similarly associated with all outcome variables, except anxiety. The pattern of associations in relation to the interaction effect of RA-by-sex also remained largely unchanged, except for conduct problems, which also became significantly associated with the interaction effect, indicating that the association between RA and conduct problems, similar to that with alcohol use, was stronger among girls. These results are presented in Supplementary Table S1.

## 4 Discussion

The present study extends the literature on RA in a non-Western society by demonstrating that higher levels of RA are associated with a broad constellation of clinically relevant mental health outcomes in adolescence, including depressive symptoms, somatic complaints, posttraumatic stress symptoms, conduct problems, as well as alcohol use, and functional impairment. Importantly, these associations emerged in a large, population-based sample of adolescents and remained significant in a multivariate path model that accounted for age, sex, socioeconomic status, and intercorrelations among the symptom domains. Together, these findings underscore RA as a marker of broad psychosocial vulnerability rather than narrowly defined interpersonal behavior. Sex differences were examined within the full multivariate model through covariate adjustment and the use of formal RA-by-sex interaction terms, allowing direct tests of whether associations differed between boys and girls. RA was more strongly associated with internalizing and stress-related symptoms among boys, whereas its association with alcohol use was stronger among girls.

Consistent with prior research, RA was robustly associated with both internalizing and externalizing difficulties (Zimmer-Gembeck et al., 2005; Card et al., 2008). The present findings extend this work by demonstrating significant associations between RA and posttraumatic stress symptoms and somatic complaints—outcomes that have received comparatively less attention in the RA literature despite their substantial developmental and public health relevance. Notably, the strongest associations among outcomes were observed between depressive symptoms and posttraumatic stress, and between depressive symptoms and somatic complaints, reflecting well-documented patterns of internalizing comorbidity in adolescence, wherein affective and stress-related symptoms frequently cluster and share common underlying vulnerabilities (Angold et al., 1999; Kessler et al., 2005; Cummings et al., 2014).

The observed association between RA and depressive symptoms is consistent with quantitative evidence that RA has a unique association with internalizing problems across childhood and adolescence (Card et al., 2008). Extending this work, the present findings show that RA remains associated with depressive symptoms even when multiple co-occurring symptom domains are modeled simultaneously. At the same time, some adolescent studies have not found a unique RA–depression association once co-occurring factors (e.g., peer victimization) are included in the same model (Prinstein et al., 2001), underscoring the notion that covariate selection and the analytic approach adopted can influence observed effects. Nonetheless, the persistence of the association in our multivariate model points to a robust link in this cohort.

The association between RA and posttraumatic stress symptoms is particularly noteworthy. Although RA is often conceptualized as a form of aggression enacted by the individual, engagement in relationally aggressive behaviors frequently occurs within volatile peer environments characterized by retaliation, exclusion, and social instability. Such contexts may expose adolescents to repeated experiences of interpersonal threat, humiliation, and loss of social safety, which are increasingly recognized as potent contributors to trauma-related symptomatology, even in the absence of discrete Criterion A trauma (Charuvastra and Cloitre, 2008; McLaughlin et al., 2013). This interpretation is further supported by prior research in Russian adolescent samples linking interpersonal stress and aggressive dynamics to trauma-related symptoms (Isaksson et al., 2020), as well as by studies demonstrating clinically significant posttraumatic stress reactions following bullying and peer victimization in school contexts (Idsoe et al., 2012). From this perspective, RA may co-occur with stressors and as an indicator of embeddedness in high-risk peer ecologies linked to trauma-related symptom development.

Similarly, the association between RA and somatic complaints aligns with models linking chronic psychosocial stress to embodied symptom expression during adolescence. Somatic symptoms in youth are often conceptualized as manifestations of affective distress and stress dysregulation rather than indicators of underlying medical pathology (Walker et al., 1991; Stickley et al., 2013; Stickley et al., 2016). Adolescents who report higher levels of RA may also experience persistent interpersonal tension, social monitoring, and emotional arousal, which can amplify physiological stress responses and contribute to recurrent physical complaints. The co-occurrence of somatic symptoms with depressive and posttraumatic stress symptoms observed in the present study further supports the view that RA is embedded within broader patterns of dysregulated stress responding.

RA was also uniquely associated with alcohol use, even when conduct problems and demographic variables were included in the path model. This finding is consistent with evidence that adolescent substance use often emerges within peer contexts that normalize risk-taking and rule-breaking behaviors and may serve both social and affect-regulatory functions (Chassin et al., 2013). Engagement in RA may be associated with affiliation with peer groups in which alcohol use is more acceptable or instrumental for maintaining social status, particularly in cultural contexts where adolescent drinking is relatively normative. Alternatively, alcohol use may function as a maladaptive coping strategy for managing negative affect associated with relational conflict and social instability (Cooper et al., 1992).

In contrast to the other outcomes, RA was not uniquely associated with anxiety symptoms in the path analysis. This non-significant result is theoretically informative. Anxiety symptoms often share substantial variance with depressive and posttraumatic stress symptoms, and it is likely that the bivariate association between RA and anxiety observed in prior studies reflects this shared internalizing distress rather than a direct relationship with RA per se (Card et al., 2008). When depressive and trauma-related symptoms are modeled simultaneously, RA’s specific contribution to anxiety may be statistically absorbed by overlapping affective and stress-response processes leaving little unique variance attributable to RA (Angold et al., 1999). This pattern suggests that RA may be more closely linked to distress processes characterized by mood dysregulation, interpersonal threat, and stress reactivity than to anxiety-specific processes such as anticipatory worry or avoidance. Meta-analytic findings consistently demonstrate positive associations between RA and anxiety (Card et al., 2008), and the present results should therefore not be interpreted as evidence that RA is unrelated to anxiety, but rather that its association with anxiety may be indirect or subsumed within broader internalizing pathways when examined in multivariate models. Importantly, the descriptive statistics did not indicate a restricted range for anxiety symptoms, suggesting that the absence of a unique RA–anxiety association in the multivariate model is unlikely to reflect uniformly low anxiety scores in the sample.

Consistent with previous work, RA was associated with greater functional impairment across domains of daily life, including peer relationships, academic functioning, and leisure activities (Merrell et al., 2006). Functional impairment represents a particularly important outcome, as it reflects the real-world impact of emotional and behavioral difficulties beyond symptom severity alone. The present findings suggest that RA is not only associated with internal distress and problem behaviors but also with meaningful disruptions in adolescents’ capacity to function effectively in key developmental contexts. From a developmental psychopathology perspective, such peer-process disruptions may initiate or accelerate cascading processes whereby social difficulties fuel emotional distress and behavioral dysregulation, which in turn, further erode school engagement and relationship quality (Masten and Cicchetti, 2010; Dishion and Tipsord, 2011). It should be noted, however, that functional impairment in the present study was examined as a global construct using the total score of the SDQ Impact Supplement. Associations between RA and functional impairment therefore reflected overall interference across life domains rather than effects specific to individual areas such as peer relations or school functioning. Future studies may benefit from examining whether particular domains of functioning are differentially associated with RA.

Sex differences in the associations between RA and psychopathology were also informative. Although girls reported higher overall levels of depressive symptoms, anxiety, somatic complaints, posttraumatic stress, and functional impairment, and boys reported more conduct problems, sex was not associated with mean levels of RA. Moderation analyses indicated that the associations between RA and depressive symptoms, anxiety, somatic complaints, and posttraumatic stress were stronger among boys, whereas the associations between RA and alcohol use and conduct problems were stronger among girls. This pattern highlights the distinction between sex differences in mean symptom levels and sex differences in the psychological correlates of RA (Archer, 2004; Card et al., 2008). One possible interpretation is that RA may be less socially normative or less expected among boys; therefore, boys who engage in relationally aggressive behaviors may experience greater social sanctions, interpersonal conflict, or internal distress related to violating gendered expectations for aggression and peer behavior (Underwood, 2003; Rose and Rudolph, 2006). This may help explain why RA had stronger associations with internalizing and stress-related symptoms among boys. By contrast, the stronger associations between RA and alcohol use and conduct problems among girls may suggest that RA is a more specific marker of externalizing vulnerability in girls than in boys. In boys, alcohol use and conduct problems may be more strongly linked to broader externalizing tendencies, overt aggression, impulsivity, or deviant peer contexts, making RA less uniquely predictive. Among girls, however, RA may be more closely embedded in peer conflict, social status competition, emotional dysregulation, and interpersonal strain, which may increase vulnerability to both conduct-related problems and substance use. Alcohol use may function as a means of coping with relational stress or as a form of social currency within peer networks in which RA is salient (Cooper, 1994; Mayeux et al., 2008). Alternatively, heightened negative affect may increase RA, conduct-related difficulties, and alcohol use among girls, consistent with affect-regulation models of adolescent substance use (Wills and Filer, 1996; Hussong et al., 2001). These interpretations remain speculative and should be tested directly in longitudinal and multi-informant studies.

No interactions were observed between RA and age, suggesting that RA’s unique associations with mental health outcomes and functional impairment were relatively stable in this cohort. This stability contrasts with some prior work suggesting developmental shifts in the social meaning of RA (Zimmer-Gembeck et al., 2005) and may reflect cultural factors or the hypothetical salience of relational stress across adolescence. Age-related main effects followed expected developmental trends in relation to mental health problems, with older adolescents reporting more depressive symptoms, somatic complaints, conduct problems, alcohol use, and functional impairment, and younger adolescents reporting higher anxiety, consistent with developmental changes in social-evaluative concerns, mood regulation, and risk-taking behavior (Steinberg, 2008; Twenge et al., 2019).

Several limitations of the current study should be acknowledged. The cross-sectional design precludes causal inference, and associations between RA and psychopathology are likely bidirectional. Exclusive reliance on self-reports may introduce shared method variance, although the self-report method is particularly well-suited for capturing covert relational behaviors and internal states. Further, a direct measure of social desirability was not included in the study. Although anonymous data collection procedures may have reduced response bias, the potential influence of social desirability, particularly in the underreporting of symptoms, cannot be ruled out. The measure of RA focused on behavior toward a disliked peer and may not fully capture RA within close friendships or digital contexts, where relational manipulation is increasingly prevalent (Kowalski et al., 2014). Although physical (overt) aggression was not explicitly controlled and some effects may reflect shared variance across aggression forms, prior research demonstrates that RA uniquely predicts internalizing symptoms and psychosocial impairment even when physical aggression is accounted for, indicating that the present findings are unlikely to be attributable solely to general aggression tendencies (Card et al., 2008). Another limitation is the relatively limited age range of the sample, which may have reduced statistical power to detect developmental moderation effects or obscured more fine-grained age-specific shifts in the functions and consequences of RA. This may have contributed to the absence of observed RA-by-age interactions. In addition, classroom or school-level clustering was not modeled; although the large sample size mitigates some concerns, failure to account for nesting may modestly bias standard errors and should be addressed in future multilevel analyses. Finally, generalizability may be limited to similar sociocultural contexts.

Despite these limitations, the present findings have important implications. They suggest that RA should be viewed not only as a social behavior problem but also as a potential indicator of broader mental health vulnerability and functional impairment. Early identification of relationally aggressive behaviors may help flag adolescents at risk for internalizing distress, trauma-related symptoms, and substance use. Interventions targeting RA may therefore benefit from incorporating social–emotional components, addressing emotion regulation, stress management, and peer relationship skills, and attending to sex-specific pathways of risk. Such approaches may reduce the multi-domain burden associated with RA and mitigate its impact on everyday functioning during this pivotal developmental period.

## Data Availability

The raw data supporting the conclusions of this article will be made available by the authors, without undue reservation.
