# Supplementary material for: Effects of sex and age on the association of relational aggression with mental health problems and functional impairment in adolescents
Source: Front Psychol. 2026 Jun 9;17:1800051. doi: 10.3389/fpsyg.2026.1800051 (PMC13289672; doi:10.3389/fpsyg.2026.1800051)
Supplement: Supplementary file 1 [file Table_1.pdf]

Supplementary TABLE 3. Path analytic model of associations between psychopathology and RA, age, and sex, with standardized regression coefficients (β) after applying square root transformation.

Parameter estimated	Depression	Anxiety	Somatic symptoms	Posttraumatic stress	Conduct problems	Alcohol use	Functional impairment
	β	β	β	β	β	β	β
RA	.13 ***	.05	.09 ***	.20 ***	.38 ***	.26 ***	.22 ***
Sex (male)	-.63 ***	-.52 ***	-.49 ***	-.72 ***	.34 **	.37 **	-.18
Age	.10 ***	-.04 *	.04 *	.02	.18 ***	.23 ***	.07 ***
SES proxy measure	-.04 *	-.04	-.03	-.06 **	-.01	.02	-.03
RA x sex	.46 ***	.36 **	.37 **	.56 ***	-.27 *	-.35 **	.10
R^2	.09	.04	.05	.12	.16	.11	.07

NOTE: RA = Relational aggression; SES = socioeconomic status; CI = confidence interval; R^2 = coefficient of determination
*** p<.001; ** p<.01; * p<.05
Names of specific assessments are provided in the Measures section.
